# Magnitude and severity of rebound pain after resolution of peripheral nerve block and associated factors among patients undergoes surgery at university of gondar comprehensive specialized hospital northwest, Ethiopia, 2022. Longitudinal cross-sectional study

**DOI:** 10.1016/j.amsu.2022.104915

**Published:** 2022-11-18

**Authors:** Belete Muluadam Admassie, Biresaw Ayen Tegegne, Wudie Mekonnen Alemu, Amare Belete Getahun

**Affiliations:** Department of Anesthesia, College of Medicine and Health Sciences, University of Gondar, Gondar, Ethiopia

**Keywords:** Rebound pain, Regional anesthesia, Peripheral Nerve block

## Abstract

**Background:**

Rebound pain is extreme pain that persists after the effects of regional anesthesia wear off. Rebound pain occurrence and intensity are influenced by patient, surgical, and anesthesia-related factors. The incidence and severity of rebound pain after peripheral nerve block resolution are both reduced by the use of perioperative multimodal strategy. The purpose of the current paper was to evaluate the frequency, seriousness, and risk factors for rebound pain following peripheral nerve block resolution.

**Method:**

A cross-sectional study centred on 384 patients who had received peripheral nerve blocks was carried out from August 20, 2021, to June 30, 2022. A semi-structured questionnaire was used to gather information within 24 h following the block's performance. SPSS 25 was used to enter and analyze the data. The change from well-controlled pain while the block is operating to severe pain within 24 h of block performance is known as rebound pain. Both univariate and multivariable analyses were used to examine the relationship between various parameters (patient, surgical, and anesthetic-related factors) and rebound pain. In the multivariable analysis, a P-value of 0.05 or lower is regarded as statistically significant.

**Results:**

The incidence of rebound pain after peripheral nerve block was resolved was 61.7% (95% CI: 56.5–66.7) with a mean rebound pain score of 4.19 ± 2. Of the total 237, 120(50.6%) had severe rebound pain after the peripheral nerve block was resolved. The use of preoperative intravenous dexamethasone (AOR: 2.6, 95%CI: 20.29–24.57), preoperative pain (AOR: 3.9, 95%CI: 41–57.4), type of surgery (AOR: 6.5, 95%CI: 1.45–11.7), post-operative NSAID (AOR: 2.2, 95%CI: 17.69–20.8), and opioid use (AOR: 2.2, 95%CI: 19.1–22.56) were independent risks associated with rebound pain.

**Conclusions:**

and Recommendation: Rebound pain occurred in 61.7% of patients and had independent associations with preoperative pain, dexamethasone premedication, type of surgery, use of an adjuvant, use of postoperative opioids, and NSAIDs. Therefore, clinicians should continue to use preventative strategies, especially for patients at higher risk of experiencing rebound pain.

## Highlights

• Incidence of rebound pain was 61.7%.
• Preoperative pain, premedication with dexamethasole, type of surgery, use of adjuvant, and postoperative analgesia were factors for rebound pain.
• It is recommended to use preventative strategies.

## 1 Introduction

### 1.1 Statement of the problem

Regional anesthesia, specifically peripheral nerve blocks (PNBs), is routinely performed for perioperative analgesia and anesthesia in patients undergoing surgery [1]. It plays a great role in maximising post-operative pain control while minimising opioid consumption and allowing a fast hospital discharge [2,3].

Rebound pain (RP) is mechanical–surgical pain caused by unopposed nociceptive inputs that are brutally uncovered after PNB resolution [3] and characterised by sudden, significant pain following regional nerve blockade regression [4]. There is a quantifiable difference in pain scores when the block is working versus the increase in acute pain encountered during the first few hours after the effects of perineurally single-injection or continuous infusion local anaesthetics resolve [5]. It may reduce or even negate the overall benefits of a peripheral nerve block [2].

The incidence of rebound pain after peripheral nerve block (PNB) resolves could reach around 40% of patients and may be due to abnormal spontaneous C-fiber hyperactivity and nociceptor hyper-excitability without mechanical nerve lesion [3]. Even in a previous study in Canada overall incidence of rebound pain after PNB regression was 49.6% [6].

Patient-related factors such as severe pre-operative pain, age less than 60 years old, female sex, and psychosocial factors such as catastrophic pain perception and depression have all been found to have a significant impact on the occurrence and severity of rebound pain [4,[7], [8], [9]].

Surgical factors can produce an abnormal level of plasticity at the peripheral nociceptor level and in the central neurons involved in receiving and processing direct and indirect inputs [8]. Damage to the peripheral nociceptor provokes a continuous firing of pain signals, leading to either an exaggerated response to normally painful stimuli or a noxious response to normally non-painful stimulation [3].

Poorly managed postoperative pain can result in adverse consequences, including impaired quality of recovery, opioid dependence, PPSP, and increased medical costs [2]. Therefore it is important to examine if rebound pain may have a significant impact on other health-related outcomes [10].

Postoperative pain is one of the most feared surgical complications reported by patients, which is frequently followed by a painful recovery. Appropriate treatment of acute postoperative pain is associated with better clinical outcomes, while inadequate pain control may negatively impact patients' postoperative experience [4].

Some strategies used to prevent and manage rebound pain, like continuous PNB catheter techniques, using local anesthetic adjuvant, multimodal analgesics, and preoperative education and counselling regarding rebound pain were effective in preventing and managing rebound pain [2,11].

Due to the shortage of experimental and clinical studies, the incidence of the rebound pain phenomenon is still poorly documented. Nevertheless, its occurrence has been increasingly reported by researchers, and it could profoundly impact the patient's recovery experience [1].

The occurrence of rebound pain may outweigh the benefits of PNBs and represent a clinically relevant problem [2].

Rebound pain is still a poorly understood concept, and few studies have evaluated its full impact on the use of regional anesthesia as a strategy to reduce long-term pain and opioid consumption. Rebound pain is a common, yet under-recognized, acute increase in pain and severity after a peripheral nerve block (PNB) has receded, typically manifesting within 24 h after the block was performed. Despite economic pressure and the well-known early benefits of PNBs, rebound pain unanswered questions are one more challenge in the area of perioperative management. Therefore, this study aimed to assess incidence, severity, and factors associated with rebound pain after peripheral nerve block is resolved.

## 2 Methodology

Ethical clearance was obtained from the institutional ethical review committee. The aim of the study was explained to each study participant, and informed consent was obtained. This study was registered with the UIN research registry (8161) and was reported in accordance with STROCSS criteria [12].

### 2.1 Study design, study setting, and population

An institution-based, longitudinal cross-sectional study was conducted from August 20, 2021, to June 30, 2022, at the PACU, recovery room, and wards. In this study, we included both elective and emergency procedures in patients who underwent an operation under peripheral nerve block alone or in combination with general anesthesia in the study period. However, patients who were lost to follow-up and Patients with a primary block failure were excluded from the study.

### 2.2 Operational definitions

**Pain-** Pain is defined as An unpleasant sensory and emotional experience associated with actual or potential tissue damage, or described in terms of such damage.

**Rebound pain-**defined as transient acute postoperative pain within 12–24hrs that ensues following resolution of sensory blocked [6].

**Rebound pain score-**the lowest pain score during the first 12 h before the PNB wears off is subtracted from the highest pain score during the first 12 h after the PNB wears off [13].

### 2.3 Sample size determination

To determine the sample size, the single population proportion formula was used. Since there was no previous study done similar to this topic, we took a proportion of 50% by assuming a 95%CI with a 5% margin of error, and finally, the sample size for the study was calculated as:

d=zα2(pqn)1/2n=(zα2)2×(pq)d2

$$\begin{array}{cc} n={\left(1.96\right)}^{2}\times \frac{\left(0.5\times 0.5\right)}{{\left(0.05\right)}^{2}} & n=384 \end{array}$$

### 2.4 Sampling method

Study participants will be selected using a consecutive sampling technique.

### 2.5 Data collection process

Before data collection, training was given to data collectors. The data collection procedures included chart review, interview, and direct measurement of the pain score after peripheral nerve block resolved using NRS within 24 h of the block performed. The questionnaire was prepared in the English language. The questionnaire includes sociodemographic variables, anesthesia, and surgical-related risk factors. To ensure the quality of data, pretesting of the data collection tool was conducted on 20 patients, or 5% of the study sample size. Data collectors were provided adequate information regarding the questionnaires. The data collectors was closely monitored by the principal investigator throughout the study period. The collected data were checked for completeness, accuracy, and clarity on the day of data collection before being entered into the database by the principal investigator. A total of two BSC anaesthetists participated in the data collection process.

### 2.6 Data analysis and interpretation

The data was entered and analyzed with SPSS version 20. Descriptive statistics were used to explain to the study participants about study variables and were presented as mean and standard deviation. Rebound pain score was approximately normal in sample distribution and means (95% confidence interval [CI]) were used to report for each variable subgroup. Univariate comparisons were analyzed by logistic regression for dichotomous outcomes. The linearity of the continuous variables concerning the logit of the dependent variable rebound pain was assessed. Univariate linear regression and multivariable analysis were performed to analyze the association of variables with the RPS.

## 3 Results

### 3.1 Sociodemographic characteristics

A total of 384 patients were included, with a mean age distribution of 30.8 ± 5.8. From a total of 384 patients, 237 (61.7%) patients developed rebound pain after resolution of peripheral nerve block with a mean rebound pain score of 4.19 ± 2.1. Most of the participants (70.8) were male (Table 1).

**Table 1:** Cross-tabulation of sociodemographic characteristics of the study participants.(N = 384).

Variables		Rebound pain frequency%			Mean RPS (95%CI)
		Overall	Yes (n = 237)	No (n = 147)	
		Frequency	237(61.7%)	147(38.7%)	
Gender:	Male	272	174(64%)	98(36%)	4.19 ± 2.1[3.94,4.5]
	Female	112	63(56.2%)	48(42.8%)	4.08[3.79,4.36]
BMI:	18.5–24.5	263	157(59.7%)	106(40.3%)	4.49[3.85,5.09]
	24.6–29.5	84	72(85.7%)	12(14.3%)	3.69[3.39,4.03]
	29.6–35	37	8(21.6%)	29(78.4%)	5.1[4.65,5.63] 6[5,6.85]

## 4 Preoperative risk factors

As the distribution of preoperative risk factors showed, all patients were on ASA I and ASAII, 376 (97.9%), and 8 (2.1%) respectively. The majority of patients who had no history of coexisting disease were 352 (91.6%). Preoperative pain was experienced by the majority of patients 237 (61.7%), with 119 (30.9%) experiencing severe pain and 194 (50.5%) receiving preoperative analgesia. Of the total 384,259 patients, 67.4% were premedicated with dexamethasone(Table 2).

**Table 2:** Cross-tabulation of the preoperative factors for rebound pain after PNB resolved. (N = 384).

Variables(n = 384)		Rebound pain frequency(%)			Mean RPS (95%CI)
		Overall	Yes n = 237	No = 102	
		Frequency	237(61.7%)	147(38.7%)	4.19 ± 2.1[3.94,4.5]
Coexisting	Yes	32	32(100%)	0(0%)	3.97[3.20,4.69]
	No	352	205(58.2%)	147(41.8%)	4.22[3.94,4.51]
ASA	I	376	229(60.9%)	147(39.1%)	4.17[3.89,4.44]
	II	8	8(100%)	0(0%)	4.62[3.33,6.0]
Preoperative pain:	Yes	237	213(89.9%)	24(10.1%)	4.43[4.15,4.71]
	No	147	69(46.9)	78(53.1)	2.0[1.69,2.33]
Severity of preoperative pain:	Mild	16	16(100%)	0(0%)	1.81[1.43,2.20]
	Moderate	147	117(79.6%)	30(20.4%)	4.86[4.53,5.21]
	Sever	119	80(67.2%)	39(32.8%)	4.3[3.86,4.70]
Preoperative analgesia:	Yes	194	104(53.6%)	90(46.4%)	3.26[2.89,3.65]
	No	190	133(70%)	57(30%)	4.95[4.60,5.25]
Dexamethasone premedication:	Yes	259	120(46.3%)	139(53.7%)	3.15[2.78,3.45]
	No	125	117(93.6%)	8(6.4%)	5.35[5.04,5.66]

## 5 Intraoperative and postoperative risk factors

Among patients who underwent an operation, bone(orthopedics) surgeries were mostly procedures, 235 (61.2%) of those 149 (38.8%) operated under digital peripheral nerve block. Of the total 243 (63.3%) who took adjuvant during nerve block, of those, 160(41.7%) were lidocaine (Table 3).

**Table 3:** Cross-tabulation of the intraoperative and postoperative factors and their association with rebound pain after PNB resolved. (N = 384).

Variables		Rebound pain frequency(%)			Mean RPS (95%CI)
		Overall	Yes n = 237	No = 102	
		Frequency	237(61.7%)	147(38.7%)	4.19 ± 2.1[3.94,4.5]
Type of surgery:	Soft tissue	149	40(26.8%)	109(73.2%)	3.37[2.74,4.02]
	Bone	235	197(83.8%)	38(16.2%)	4.36[4.07.4.60]
Surgical site:	Upper limb	300	216(72%)	84(28%)	4.41[4.14,4.69]
	Lower limb	84	21(25%)	63(75%)	1.86[1.5,2.19]
General Anesthesia:	Yes	78	31(39.7%)	47(60.3%)	2.2[1.90,2.43]
	No	306	206(67.3%)	100(32.7%)	4.54[4.25,4.80]
Type of PNB:	Interscalene	61	61(100%)	0(0%)	5.24[4.72,5.72]
	Supraclavicular	90	90(100%)	0(0%)	4.39[4.0,4.77]
	Lumbar plexus	42	5(11.9%)	37(88.9%)	1.6[0.5,2.5]
	Femoral compartment	42	16(38.1%)	26(61.9%)	1.94[1.59,2.31]
	Digital peripheral NB	149	65(43.6%)	84(56.4%)	3.71[3.24,4.23]
	Type of LA: Bupivacaine	384	237(61.7%)	147(38.3)	4.19[3.92,4.45]
Adjuvant used:	Yes	243	108(44.4%)	135(55.6%)	2.64[2.39,2.90]
	No	141	129(91.5%)	12(8.5%)	5.55[5.27,5.84]
Type of Adjuvant:	Opioid	83	48(58.3%)	35(42.2%)	2.45[2.15,2.79]
	Lidocaine	160	60(37.5%)	100(62.5%)	2.8[2.4,3.21]
Post-operative opioid:	Yes	113	109(94.5%)	4(5.5%)	4.84[4.50,5.20]
	No	271	128(47.2%)	143(52.8%)	3.62[3.27,3.97]
Post op NSAID:	Yes	114	102(89.5%)	12(10.5%)	5.3[5,5.56]
	No	270	135(50%)	135(50%)	3.4[3.03,3.76]
Post op paracetamol:	Yes	8	8(100%)	0(0%)	5.63[4.86,6.4]
	No	368	221(60.1%)	147(39.9%)	4.3[3.98,4.52]
Duration of surgery mean ± SD (2.87 ± 1.26):	≤2.87	78	65(36.5%)	113(63.5%)	3.46[2.95,3.94]
	>2.87	206	172(83.5%)	34(16.5%)	4.49[4.22,4.80]
Duration of motor block mean ± SD (5.81 ± 1.54):	≤5.81	66	136(81.9%)	30(18.1%)	3.9[3.53,4.29]
	>5.81	218	101(46.3%)	117(53.7%)	4.58[4.21,4.98]
Duration of sensory block mean ± SD (9.54 ± 2.9):	≤9.54	08	167(80.3%)	41(19.7%)	4.13[3.84,4.44]
	>9.54	176	70(39.8%)	106(60.2%)	4.34[3.84,4.84]
The volume of LA mean ± SD (34.4 ± 7.8):	>34.4	44	69(47.9%)	75(52.1%)	2.92[2.46,3.37]
	≤34.4	240	168(70%)	72(30%)	4.74[4.45,5.05]
Dose of LA mean ± SD (85.5 ± 19.7):	>85.5	161	86(53.4%)	75(46.6%)	2.94[2.57,3.32]
	≤85.5	223	151(67.7%)	72(32.3%)	4.92[2.46,3.37]
The severity of Rebound pain					
Mild		88(36.5%)			2.03[1.87,2.26]
Moderate		33(13.7%)			4.21[3.82,4.62]
Sever		120(49.8%)			5.8[5.55,6.06]

## 6 Magnitude of rebound pain after PNB resolved

**Fig. 1:**
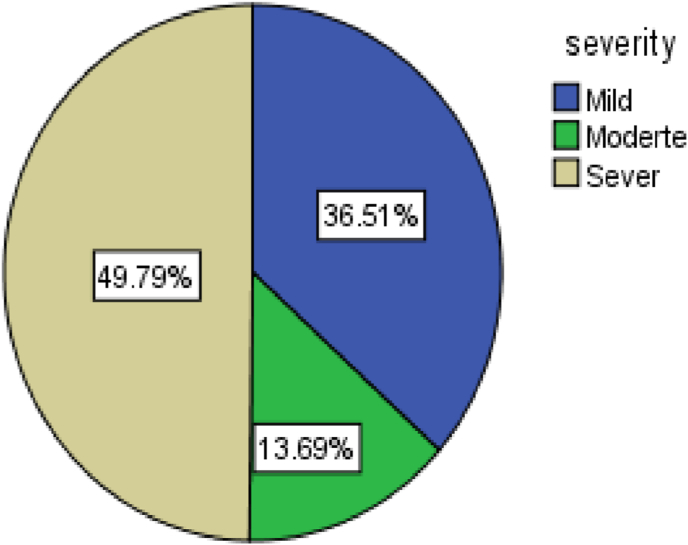
Severity of rebound pain after rebound pain resolved.(N = 384).

The magnitude of rebound pain after resolution of peripheral nerve block was 237 (61.7%) (95%CI: 56.5–66.7)with a mean rebound pain score of 4.19 ± 2. Of the total 237, 120(50.6%) had severe rebound pain after PNB resolved(Fig. 1).

## 7 Factors associated with rebound pain after peripheral nerve block resolved

In the univariate logistic regression analysis, age and sex of the participants, having coexisting, having preoperative pain, premedication with dexamethasone, preoperative analgesia is given, type and site of surgery, having supraclavicular nerve block, use of an adjuvant, duration of surgery, the volume of local anaesthetics, and use of postoperative opioids and NSAIDs were significant at *p*-value <0.2. However, having preoperative pain, premedication with dexamethasone, type of surgery, use of an adjuvant, and use of postoperative opioids and NSAIDs were significantly associated with rebound pain in multivariable analysis at which *p*-value was <0.05. Participants who did not receive preoperative analgesia were 3.8 times (AOR: 3.8, 95%CI: 19.9–23.1), more likely to develop rebound pain when compared to those who had received preoperative analgesia. Similarly, those patients having preoperative pain were 3.9 times (AOR: 3.9, 95%CI: 41–57.4) more likely to develop rebound pain when compared to those who had no preoperative pain. Those patients premedicated with dexamethasone were 2.6 times(AOR: 2.6, 95%CI: 20.29–24.57), less likely to develop rebound pain compared to those not premeditated. Patients who underwent bone surgery 6.5times (AOR: 6.5, 95%CI: 1.45–11.7), were more likely to develop rebound pain compared to those who underwent soft tissue surgery. use of adjuvant for peripheral nerve block 0.4 times(AOR: 0.4, 95%CI: 18.37–19.9), less likely to develop rebound pain compared to nerve block without adjuvant. Patients who take postoperative opioids and NSAIDs were less likely to develop rebound pain with AOR: 2.2, 95%CI: 19.1–22.56 and AOR:2.2,95%CI, 17.69–20.8 compared to those not take postoperative analgesia respectively (Table 4, Table 5, Table 6).

**Table 4:** Univariate logistic regression analysis of patient characteristics and preoperative factors for association with incidence of rebound pain after PNB resolved. (N = 384).

Variable	Reference group	OR	95%CI	p-value
Age of participants	–	0.28	0.74–0.183	0.001
Sex of participants	Male	0.85	0.197–1.01	0.18
ASA physical status	One	0.03	−1.04–1.93	0.55
Having co-existing disease	No	3.7	2.22–5.21	0.002
Having Preoperative pain	No	6.86	5.91–7.815	0.01
The severity of preoperative pain				
Mild	Mild	0.01	2.3–6.1	0.52
Moderate		0.7	1.25–5.2	0.34
Sever		2.339	0.96–3.7	0.01
Taking preoperative analgesia	Yes	1.68	1.19–2.17	0.004
Premedication with dexamethasone	Yes	2.2	1.79–2.69	0.017

**Table 5:** Univariate logistic regression analysis of patient characteristics and preoperative factors for association with incidence of rebound pain after PNB resolved. (N = 384).

Variable	Reference group	OR	95%CI	p-value
Type of surgery	Soft tissue	0.17	0.29–1.69	<0.001
Site of surgery	Lower limb	6.96	5.96–7.96	0.001
General anesthesia	No	0.4	1.69–3.06	0.74
Type of PNB	Interscalene	2.3	1.06–3.68	0.06
Supraclavicular		1.7	1.55–2	<0.001
Lumbar plexus		0.2	1.2–3.1	0.13
Femoral compartment		1.2	0.25–1.2	0.62
Digital peripheral NB		1.3	1.3–2.52	0.15
The dose of LA used	–	0.42	0.038–0.67	0.27
Adjuvant used	Yes	0.69	2.52–3.29	0.01
Type of adjuvant	Opioid	0.12	0.17–0.86	0.28
Duration of surgery	–	0.13	0.017–0.45	0.035
Duration of sensory block	–	0.09	−0.036–0.22	0.35
Duration of motor block	–	0.091	−0.042–0.26	0.26
Post-op opioid used	Yes	1.22	0.71–1.73	0.002
Post-operative NSAID used	No	−0.45	−2.39- - 1.42	0.001
Post-op PCM used	No	−0.122	−2.88–0.68	0.61
BMI	18.5–24.5	0.2	0.31–1.33	0.002
Dose category	Above mean	0.45	0.128–1.79	0.024
Volume category	Above mean	0.399	1.289–2.35	0.022
Duration of surgery	Below mean	0.22	0.45–1.6	<0.001
Duration of sensory block	Above mean	0.04	3.11–4.7	0.48
Duration of motor block	Above mean	0.27	0.14–1.2	0.34

**Table 6:** Multivariable Logistic Regression with rebound pain after PNB resolved. (N = 384).

Variables	Ref group	AOR	95%CI	P-value
Having coexisting disease	No	4.1	16.46–19.67	0.13
Having preoperative pain	Yes	3.9	41–57.4	0.01
Premedication with dexamethasone	Yes	2.6	20.29–24.57	0.008
Pre-operative analgesia given	Yes	3.8	19.9–23.1	0.01
Type of surgery	Soft tissue	6.5	1.45–11.7	0.04
Adjuvant used	Yes	0.4	18.37–19.9	0.01
Duration of surgery	Below mean	2.8	53.8–58.6	0.06
Postoperative opioid used	Yes	2.2	19.1–22.56	0.002
Postoperative NSAID used	Yes	2.2	17.69–20.8	0.001

## 8 Discussion

Rebound pain is a common, yet under-recognized, acute increase in pain severity after a peripheral nerve block (PNB) has receded, typically manifesting within 12–24 h after the block was performed and adversely affecting sleep quality [14]. The incidence of the rate of rebound pain could reach 40% of patients at PNB resolution [3].

The current paper was conducted to find out the magnitude, severity, and factors associated with rebound pain after resolution of peripheral nerve block. The overall incidence of rebound pain after peripheral nerve block was resolved was 61.7%(95% CI: 56.5–66.7) with a mean rebound pain score of 4.19 ± 2.1[95% CI: 3.94, 4.5].

A retrospective cohort study done in Canada showed that the incidence of rebound pain after PNB was resolved was 49.6%. This is relatively low when compared with current study. The possible explanation of this discrepancy could be the study design, sample size, and technique of peripheral nerve block [6]. In addition, a previous comparative study done in @New-York stated that a single injection had a higher risk of rebound pain compared to continuous peripheral nerve block [15]. However, in present work, all PNB done in a single injection could be the cause for higher incidence.

A prospective study carried out in Belgium found that the incidence of rebound pain after peripheral nerve block reached up to 40% [3]. Our finding is relatively higher than the above study. The possible explanation for the high magnitude of rebound pain in the current paper might be due to the small sample size and could be due to clinical set-up differences, and techniques of nerve block. In addition, in the present work all PNB was done under blind landmark and nerve stimulator technique. This might have caused mechanical and chemical nerve erosion/insult caused by PNB.

In the current paper, preoperative intravenous dexamethasone use was 2.6 times (AOR: 2.6, 95%CI: 20.29–24.57), less likely to develop rebound pain than those who had not taken intravenous dexamethasone. This present work is supported by previous studies in which using intravenous dexamethasone decreased the risk of rebound pain after PNB resolved or had a significant association with rebound pain [6]. Dexamethasone has been shown to prolong PNB duration when given perineurally compared with intravenously, although a recent systematic review showed that either route is equivalent in terms of duration of block analgesia, 24 h pain scores, and cumulative opioid consumption at 24 h postoperatively [16,17]. Dexamethasone at single doses greater than 0.1 mg/kg has been shown to reduce postoperative pain in a previous meta-analysis [18]. The reduction in rebound pain incidence and RPS found may be consistent with the known effect of iv dexamethasone on postoperative pain in general rather than any possible effect on PNB duration [6].

In the present study, those patients having preoperative pain were 3.9 times (AOR: 3.9, 95%CI: 41–57.4), more likely to develop rebound pain than those who had no preoperative pain. This might be supported by preoperative pain level was a significant predictor of severe postoperative pain in several studies across a variety of non-cardiac surgery [8,19]. Preoperative pain may be associated with rebound pain [[6], [7], [8],^20^]. This is also supported by the fact that patients with pre-existing joint pain who were more likely to report rebound pain following the use of PNB in total hip or knee arthroplasty [20].

In present work, patients who did not receive preoperative analgesia were 3.9 times (AOR: 3.9, 95%CI: 41–57.4) more likely to develop rebound pain when compared to those who had received preoperative analgesia. This might be explained by having preoperative analgesia as preemptive or preventive analgesia that decreases peripheral and central sensitization [21,22].

In the present work, patients who underwent bone surgery 6.5 times (AOR: 6.5, 95%CI: 1.45–11.7), were more likely to develop rebound pain compared to those who underwent soft tissue surgery. The current paper was supported by a previous study done in Canada that showed that patients having bone surgery had a significant association with rebound pain [6,8].

In the present work, the use of adjuvant for peripheral nerve block was 0.4 times(AOR: 0.4, 95%CI: 18.37 19.9), less likely to develop rebound pain compared to nerve block without adjuvant. This might be explained by a previous study [23] adding adjuvant on local anaesthetics in addition to prolonging the duration of analgesia, it helps to reduce overall dose requirements for local anaesthetics could decrease the incidence of rebound pain after PNB is resolved. In contradictory to a study done in Canada [24] in current paper, gender of the patients has no significant association with the occurrence of rebound pain. This finding supported by a previous study done in the Netherlands on predictors of postoperative pain [8].

In the present work, patients who received postoperative analgesia like opioids and NSAIDs were less likely to experience a rebound after PNB was resolved. This might be explained by using perioperative multimodal analgesia to decreases perioperative opioid use, which has an opioid sparing effect and also decrease the severity of postoperative pain [25,26].

## 9 Strength and limitation

The current paper's diversity of factors studied for association with rebound pain, potentially representing the largest single investigation on rebound pain, was considered the study's strength. Present work does not assess the specific time for which maximal rebound pain occurs, and the effect of continuous peripheral nerve block on rebound pain and severity is also not assessed.

## 10 Conclusion

The overall magnitude of rebound pain after PNB resolved was 61.7%, and patients having preoperative pain, premedication with dexamethasone, type of surgery, use of an adjuvant, use of postoperative opioids, and NSAIDs were independent factors associated with rebound pain.

## Recommendations

Future research should look into the specific time of occurrence of rebound pain after PNB has been resolved, as well as the effect of continuous PNB on rebound pain.

## Ethics approval and consent to participate

Ethical clearance was obtained from the institutional ethical review committee. The aim of the study was explained to each study participant and informed consent was obtained. Anyone not volunteering for participation was informed that they had full right not to participate or stop at any time.

## Funding source

University of Gondar.

## Authors’ contributions

This work was carried out in collaboration among all authors. B.M Admassie contributed to the conception, the review, and interpreted the result. B. A Tegegne, W.M Alemu, A.B Getahun in commenting from conception till manuscript preparation.

## Registration of research studies

1. Name of the registry: research registry
2. Unique Identifying number or registration ID: 8161
3. Hyperlink to your specific registration (must be publicly accessible and will be checked): https://www.researchregistry.com/browse-the-registry#home/

## Guarantor

The Guarantor is the one or more people who accept full responsibility for the work and/or the conduct of the study, had access to the data, and controlled the decision to publish. uogbelete@gmail.com Phone number:+251945567123 p. o.box:196.

## Consent for publication

Not applicable.

## Provenance and peer review

Not commissioned, externally peer-reviewed.

## Availability of data and materials

All data generated or analyzed during this study were included in this published article and available on request.

## Declaration of competing interest

The authors declared that they have no competing interests.

## Acronyms and Abbreviation

EC: @Ethiopian Calendar
GC: @Gregorian calendar
ISB: @ Interscalene block
MI: @Myocardial ischemia
NRS: @Numerical rating scale
PCA: @Patient controlled analgesia
PNB: @peripheral nerve block
RP: @Rebound pain
RPS: @Rebound pain score
